# An Expanded Ribosomal Phylogeny of Cyanobacteria Supports a Deep Placement of Plastids

**DOI:** 10.3389/fmicb.2019.01612

**Published:** 2019-07-12

**Authors:** Kelsey R. Moore, Cara Magnabosco, Lily Momper, David A. Gold, Tanja Bosak, Gregory P. Fournier

**Affiliations:** ^1^Department of Earth, Atmospheric and Planetary Sciences, Massachusetts Institute of Technology, Cambridge, MA, United States; ^2^Center for Computational Biology, Flatiron Institute, Simons Foundation, New York, NY, United States; ^3^Department of Earth and Planetary Sciences, University of California, Davis, Davis, CA, United States

**Keywords:** cyanobacteria, Archaeplastida, chloroplast, evolution, phylogenetic tree

## Abstract

The phylum Cyanobacteria includes free-living bacteria and plastids, the descendants of cyanobacteria that were engulfed by the ancestral lineage of the major photosynthetic eukaryotic group Archaeplastida. Endosymbiotic events that followed this primary endosymbiosis spread plastids across diverse eukaryotic groups. The remnants of the ancestral cyanobacterial genome present in all modern plastids, enable the placement of plastids within Cyanobacteria using sequence-based phylogenetic analyses. To date, such phylogenetic studies have produced conflicting results and two competing hypotheses: (1) plastids diverge relatively recently in cyanobacterial evolution and are most closely related to nitrogen-fixing cyanobacteria, or (2) plastids diverge early in the evolutionary history of cyanobacteria, before the divergence of most cyanobacterial lineages. Here, we use phylogenetic analysis of ribosomal proteins from an expanded data set of cyanobacterial and representative plastid genomes to infer a deep placement for the divergence of the plastid ancestor lineage. We recover plastids as sister to *Gloeomargarita* and show that the group diverges from other cyanobacterial groups before *Pseudanabaena*, a previously unreported placement. The tree topologies and phylogenetic distances in our study have implications for future molecular clock studies that aim to model accurate divergence times, especially with respect to groups containing fossil calibrations. The newly sequenced cyanobacterial groups included here will also enable the use of novel cyanobacterial microfossil calibrations.

## Introduction

Two major groups of organisms produce oxygen by oxygenic photosynthesis: Cyanobacteria, the bacterial group in which this metabolism first evolved, and photosynthetic eukaryotes. Photosynthesis in eukaryotes is carried out by plastids, specialized organelles capable of capturing and converting light energy using Photosystems I and II. Following the first suggestion of a cyanobacterial ancestor of the plastids (Schimper, 1885), studies have attempted to characterize the commonalities and relationships between different groups of cyanobacteria and plastids. Subsequent work firmly established that plastids originated in an endosymbiotic event in which an early eukaryote engulfed a cyanobacterium (Whatley et al., 1979; Whatley and Whatley, 1981; Cavalier-Smith, 1982; Douglas, 1998). A major component of this work has attempted to determine the sequence of evolutionary events leading from the single endosymbiotic event in the ancestor of Archaeplastida to the subsequent diversification of the three primary photosynthetic eukaryote lineages (Glaucophyta, Rhodophyta, and Viridiplantae) (Whatley and Whatley, 1981; Cavalier-Smith, 1982; McFadden, 2001). However, the nature of the direct cyanobacterial ancestor of the major plastid lineages and its relationship to other cyanobacteria remain poorly understood (Sato, 2007). Cyanobacteria and plastids share several key features such as thylakoid membranes containing Photosystems I and II, but the ancestral plastid lineage is highly derived and altered from its cyanobacterial ancestor. Additionally, the ultrastructure and pigments of plastids differ among the various groups of photosynthetic eukaryotes. Consequently, structural and morphological comparisons alone cannot be used to determine the closest cyanobacterial relative to the plastid ancestor.

Phylogenetic reconstructions based on nucleotide or amino acid sequence data provide an alternative strategy for determining the evolutionary relationships and history of plastids and Cyanobacteria. However, these approaches have an independent set of challenges. Studies to date have attempted to uncover the placement of plastids within the cyanobacterial tree using a range of sequence datasets and taxonomic sampling. The topologies for the major cyanobacterial groups produced by these different analyses are in general agreement (e.g., Shih et al., 2013; Schirrmeister et al., 2015). However, the placement of plastids – and therefore the divergence of their ancestral cyanobacterial lineage – has proven contentious. Two main hypotheses emerge from these studies. Hypothesis 1 places the divergence of the plastid lineage relatively recently, close to the nitrogen fixing cyanobacteria (Douglas and Turner, 1991; Douglas, 1998; Ochoa de Alda et al., 2014). This topology was recovered using 16S rRNA sequences (Douglas and Turner, 1991), a concatenation of 16S rRNA and 23S rRNA sequences (Ochoa de Alda et al., 2014), genes such as *tufA, atpB, rpoC1*, and *psbA* (Douglas, 1998), a concatenated dataset of 16S rRNA and the *rbcL* genes (Falcón et al., 2010), and photosynthetic eukaryotic nuclear encoded protein sequences and their cyanobacterial homologs (Dagan et al., 2012). An additional study by Deusch et al. (2008) used nuclear encoded proteins in a group of photosynthetic eukaryotic lineages with cyanobacterial homologs and found that plastids are most closely related to heterocystous nitrogen-fixing cyanobacteria. Hypothesis 2 places plastids much deeper in the tree. This topology was recovered by studies that used aligned amino acid sequences from a wide range of concatenated protein and rRNA datasets (Rodríguez-Ezpeleta et al., 2005; Reyes-Prieto et al., 2010; Criscuolo and Gribaldo, 2011; Li et al., 2014). In general, many of these studies use a limited set of cyanobacteria and do not always recover the phylogenetic placement of plastids with high confidence. A more recent analysis included a newly sequenced cyanobacterium (*Gloeomargarita lithophora*) and used a concatenation of 97 proteins chosen from plastid genomes to suggest a deep placement of plastids with *G. lithophora* as a sister group to their cyanobacterial ancestor (Ponce-Toledo et al., 2017). This result was further supported by phylogenies of concatenated 16S and 23S rRNA datasets, although Bayesian consensus trees generated from these datasets did not resolve the deep relationship between *Pseudanabaena*, *G. lithophora+*plastids, and other more derived clades of cyanobacteria. Another recent study similarly recovered deep divergences of both plastids and *G. lithophora* using a concatenation of proteins for Cyanobacteria and a concatenation of 26 nucleotide sequences for the plastids (Sánchez-Baracaldo et al., 2017). Both analyses recovered a topology in which plastids diverged after *Pseudanabaena* and the clade containing *Thermosynechococcus* (Sánchez-Baracaldo et al., 2017). Overall, the range of data sets and taxon sampling used in previous studies, and the absence or differing placement of some key groups like *Pseudanabaena –* a possible sister group to the plastids (Shih et al., 2013) – suggest that establishing the placement of plastids within the cyanobacterial tree requires further investigation.

Resolving the placement of plastids is complicated by several factors that may influence phylogenetic reconstructions. For example, the transition from endosymbiont to organelle is accompanied by gene loss, endosymbiotic gene transfer, amino acid and nucleotide compositional changes, and even rearrangement of the genetic code itself (Kurland, 1992). These processes decrease the amount of phylogenetic information available for evolutionary inference and increase the chance of tree reconstruction artifacts such as long branch attraction. Such factors have been noted in debates regarding the placement of the alphaproteobacterial ancestor lineage of mitochondria (Fitzpatrick et al., 2006; Ferla et al., 2013; Wang and Wu, 2015; Martijn et al., 2018), and may impact the nucleotide or amino acid sequences used in any given phylogenetics study to varying degrees. This may, in part, explain the conflicting topologies recovered for the placement of plastids within Cyanobacteria. Another complicating factor is horizontal gene transfer (HGT), as the cyanobacterial lineage ancestral to plastids may have acquired genes from other cyanobacterial groups. This is a frequent occurrence, preferentially involving cyanobacteria-specific genes, including photosynthesis-associated genes subsequently inherited by plastids (Yerrapragada et al., 2009).

Resolving the phylogenomic history of cyanobacterial genes, including plastid genes, is a complex problem. It is entirely plausible that many plastid genes trace different deep evolutionary histories through cyanobacterial evolution. This is a well-known critique and challenge to Tree of Life studies in general (Bapteste et al., 2009). One approach, favored by several of the studies above, and consistent with many Tree of Life studies (e.g., Williams et al., 2012; Hug et al., 2016) is to use a standard “core” set of conserved and ubiquitous genes that are expected to infrequently experience HGT, and thus are more likely to trace the underlying history of cellular descent. While such a tree may not capture the totality of the complex evolution of plastid genomes, it is more likely to represent the tree of cellular descent that necessarily links the plastid to its cyanobacterial origins.

Here, we elaborate on ribosomal protein studies addressing the placement of plastids within the cyanobacterial tree. We use an updated and expanded taxonomic sampling, which includes *Pseudanabaena* and 20 previously unsequenced cyanobacterial species, to generate a plastid/cyanobacteria species tree. This expanded diversity provides a means to potentially better resolve the deep divergences within Cyanobacteria, including the ancestor lineage to plastids. The resulting phylogenies show long branches among plastids relative to crown Cyanobacteria, and place the plastids deep within the cyanobacterial tree, between *Synechococcus* sp. JA-2-3B a 2-13 and *Pseudanabaena*. These results are especially relevant to molecular clock studies that use cyanobacterial/plastid phylogenetic trees calibrated by fossil evidence, as accurate tree topologies are important for estimation of divergence times.

## Materials and Methods

### Selection of Available Sequences

We selected a core set of 36 cyanobacterial species, 32 plastid lineages, and 45 bacterial outgroups from genomes that were available on the NCBI database^1^ (Table 1). We selected between one and eleven representative taxa from each of the major cyanobacterial groups described in previous work (Schirrmeister et al., 2011; Shih et al., 2013), depending on how many genomes are currently available from those groups. We additionally selected representatives within groups that had the most complete set of ribosomal proteins used in our analyses in an effort to maximize the effectiveness of our data set and fairly compare across all groups without introducing bias in lack of sequence information. All protein analyses used a concatenation of 30 conserved large and small subunit ribosomal protein sequences (Table 2) as initially selected in Magnabosco et al. (2018), with orthologs from additional cyanobacterial and plastid genomes identified in Genbank using BLASTp (Altschul et al., 1990), taking the top reciprocal hit in each case, when present. In some cases, a homolog to the query ribosomal protein was not detected. These were left as missing data in the concatenated alignment and are noted in section “rRNA Sequence Trimming” in Supplementary Methods. Taxa were selected to provide representative coverage of the major cyanobacterial groups, and to avoid oversampling of heavily sequenced cyanobacteria such as *Prochlorococcus*.

**TABLE 1:** List of taxa included in phylogenetic trees.

**Cyanobacterial species from GenBank**	**Cyanobacterial species from enrichments and culture collections**	**Plastids from GenBank**	**Chloroflexi species from GenBank**	**Melainabacteria from GenBank**	**Bacterial outgroups from GenBank**
*Acaryochloris marina* MBIC11017 SAMN02604308	XAN 1^*^ SAMN08617393	*Acer davidii* PRJNA325991	*Anaerolinea thermophila* SAMD00061114	*Gastranaerophilus phascolarctosicola* (Zagget bin 221) M_p13725 SAMN05890510	*Anaplasma phagocytophilum* PRJNA336
*Anabaena cylindrica* PCC7122 CP003659.1	XAN 14^*^ SAMN08617394	*Adiantum capillus veneris* PRJNA12239	*Caldilinea aerophila* *SAMD00061033*	*Obscuribacter phosphatis* (Mle1_12) M_p26868 PRJNA347481	*Bacteroides fragilis* SAMN02463689
*Anabaena* sp. LE011-02 SAMN04028828	*Aphanothece cf. minutissima* CCALA015 SAMN08617395	*Amborella trichopoda* PRJNA238126	*Oscillochlorois trichoides* *SAMN02469416*	*Gastranaerophilaceae* Zag_1 (Zagget bin 1) p2523533517	*Candidatus Pelagibacter* SAMN02603690
*Arthrospira* PCC8005 SAMN08865926	*Aphanothece hegewaldii* CCALA016 SAMN08617396	*Cattleya liliputana* PRJNA356572	*Chloroflexus aurantiacus* SAMN02598539	*Gastranaerophilaceae* Zag_111 (Zagget_111_MP) M_p19866	*Capnocytophaga canimorsus* SAMEA3180108
*Arthrospira platensis* C1 PRJNA299041	*Chamaesyphon polymorphus* CCALA037 SAMN08617397	*Chara vulgaris* PRJNA19853	*Dehalococcoides mccartyi* SAMN02444487		*Cardinium endosymbiont* *SAMEA3139007*
*Arthrospira platensis* NIES 39 PRJDA42161	*Chlorogloea* CCALA695 SAMN08617398	*Chlamydomonas reinhardtii* PRJNA21061	*Dehalogenimonas lykanthroporepellens* SAMN02598529		*Caulobacter segnis* SAMN02598513
*Gloeomargarita lithophora* Alchichica-D10 GCA_001870225.1	*Chroococcidiopsis* CCALA043 SAMN08617399	*Cyanidium caldarium* PRJNA12230	*Ktedonobacter racemifer* PRJNA27943		*Cellulophaga geojensis* SAMN02952948
*Chamaesiphon minutus* PC 6805 PRJNA158825	*Chroococcidiopsis* CCALA051 SAMN08617400	*Cyanophora paradoxa* PRJNA15743	*Nitrolancetus hollandicus* SAMEA2272167		*Chlorobaculum parvum* SAMN02598460
*Chroococcidiopsis thermalis* PCC7203 SAMN02261359	*Cyanosarcina* CCALA770 SAMN08617401	*Durinskia baltica* PRJNA50277	*Roseiflexus castenholzii* SAMN02598306		*Chlorobium tepidum* SAMN02604006
*Coleofasciculus chthonoplastes* PCC7420 SAMN02436227	*Leptolyngbya frigida* ULC18 SAMN08617402	*Ectocarpus siliculosus* PRJNA41869	*Sphaerobacter thermophiles* SAMN02598446		*Chloroherpeton thalassium* SAMN02598461
*Cyanobium gracile* PCC6307 SAMN02261330	*Merismopedia glauca* CCALA99 SAMN08617403	*Emiliania huxleyi* PRJNA20387	*Thermomicrobium roseum* SAMN02603430		*Ehrlichia canis* SAMN02598261
*Cyanothece* sp. PCC7425 SAMN00000655	Phorm 6^§^ SAMN08617404	*Fistulifera solaris* PRJNA66177			*Elizabethkingia meningoseptica* SAMN02471026
*Cyanothece* sp. PCC7822 SAMN00000663	Phorm 46^§^ SAMN08617405	*Gracilariopsis lemaneiformis* PRJNA314878			*Flavobacterium aquatile* SAMN03025770
*Fischerella* PCC9065 SAMD00042785	*Phormidesmis priestleyi* ULC007 SAMN08617406	*Huperzia lucidula* PRJNA13599			*Fluviicola taffensis* SAMN00713599
*Gloeobacter kilaueensis* JS1 SAMN02604186	*Pleurocapsa* CCALA161 SAMN08617407	*Koliella corcontica* PRJNA267422			*Ignavibacterium album* SAMN02603949
*Gloeobacter violaceus* PCC7421 SAMD00061120	CCP3^†^ SAMN08617408	*Kryptoperidinium foliaceum* PRJNA50237			*Kordia algicida* SAMN02436147
*Leptolyngbya boryana* PCC6306 SAMD00079812	CCP1 ^†^ SAMN08617409	*Lilium tsingtauense* PRJNA291892			*Magnetococcus marinus* SAMN02598452
*Leptolyngbya* sp. PCC7375 SAMN02256520	CCT1^†^ SAMN08617410	*Marchantia paleacea subsp diptera* PRJDB3738			*Marivirga tractuosa* SAMN00138949
*Moorea producens* PAL SAMN05826283	CCP5 ^†^ SAMN08617411	*Mesostigma viride* PRJNA12234			*Melioribacter roseus* SAMN02603097
*Nostoc* sp. PCC7120 PRJNA244	CCP2^†^ SAMN08617412	*Nannochloropsis salina* PRJNA218133			*Methylobacterium radiotolerans* SAMN00000277
*Nostoc* sp. PCC7524 SAMN02261333	CCP4^†^ SAMN08617413	*Odontella sinensis* PRJNA12229			*Niabella soli* SAMN02261390
*Pleurocapsa minor* UAM388 PRJNA158829		*Oryza rufipogon* PRJNA45999			*Odoribacter splanchnicus* PRJNA43469
*Prochlorococcus marinus* MIT9313 SAMEA3138210		*Phaeodactylum tricornutum* PRJNA18283			*Owenweeksia hongkongensis* SAMN02261417
*Prochlorococcus marinus* str. CCMP1375 SAMN02603142		*Physcomitrella patens* PRJNA28131			*Paludibacter propionicigenes* SAMN00016731
*Prochlorococcus* sp. MIT0801 SAMN02769563		*Porphyridium purpureum* PRJNA232187			*Parvularcula bermudensis* SAMN02603918
*Prochlorothrix hollandica* PCC9006 PRJNA158811		*Pseudo-nitzschia multiseries* PRJNA287360			*Pedobacter kyungheensis* SAMN03145168
*Pseudanabaena* sp. PCC6802 SAMN02261339		*Pyropia yezoensis* PRJNA16670			*Pelagibaca bermudensis* SAMN02436105
*Pseudanabaena* sp. PCC7367 SAMN02261336		*Thalassiosira pseudonana* PRJNA344076			*Prosthecochloris aestuarii* SAMN02598281
*Rivularia* sp. PCC7116 SAMN02232049		*Thorea hispida* PRJNA20561			*Rhodospirillum centenum* SAMN02603908
*Spirulina subsalsa* PCC9445 PRJNA158827		*Ulnaria acus* PRJNA81371			*Rickettsia typhi* SAMN02603530
*Stanieria cyanosphaera* PCC7437 SAMN02261352		*Volvox carteri f nagariensis* PRJNA13109			*Rikenellaceae bacterium* SAMEA3139033
*Synechococcus elongatus* PC 6301 PRJNA13282		*Zygnema circumcarinatum* PRJNA17049			*Schleiferia thermophile* SAMN02595509
*Synechococcus* sp. JA-2-3B a 2-13 SAMN02604049					*Spirosoma linguale* SAMN00002598
*Synechococcus* sp. KORDI-49 SAMN02202184					*Wolbachia* *SAMN02604272*
*Thermosynechococcus elongatus* BP1 PRJNA308					
*Trichodesmium erythraeum* IMS101 SAMN02598485					

Taxa include species of Cyanobacteria whose genomes are available on the GenBank database and newly sequenced Cyanobacteria. Newly sequenced species were ordered from culture collections (CCALA and ULC) or enriched in the lab from environmental samples collected in Yellowstone National Park^*^ or Cape Cod^†^ by the Bosak Lab at MIT or collected in the Arctic by Dr. Anne Jungblut at London Natural History Museum^§^. Also listed are plastid and outgroup species whose genomes are available on the GenBank database.

**TABLE 2:** Large and small subunit proteins used in this study.

**Large Subunit (50S)**	**Small Subunit (30S)**
L1	S2
L2	S3
L3	S4
L4	S5
L5	S7
L6	S8
L10	S9
L13	S10
L14	S11
L15	S12
L18	S13
L22	S14
L23	S15
L24	S17
L29	S19

For ribosomal RNA datasets, available 16S rRNA sequences were obtained from each of the selected genomes through the Silva high quality ribosomal RNA database (Quast et al., 2013). 23S rRNA was additionally obtained from genomes where a 16S sequence was found. Complete rRNA gene sequences were identified in the newly sequenced cyanobacterial genomes using BLASTn (Altschul et al., 1990). In the case of rRNA gene duplicates within genomes, only one sequence was selected.

### Selection and Sequencing of Additional Cyanobacteria

In addition to cyanobacterial species with ribosomal protein sequences that are currently available on the GenBank database, our analyses included 20 previously unsequenced cyanobacteria and one genome previously sequenced and described by Lara et al. (2017) and Cornet et al. (2018) (Table 1). These were ordered from culture collections or enriched from environmental samples (See Supplementary Methods). We sequenced the genomes of these species both to increase the coverage of cyanobacteria represented in phylogenetic trees, and to improve our understanding of the phylogenetic placement of organisms with distinct morphologies, behaviors and ecological niches.

DNA was extracted using a PowerSoil DNA Isolation Kit (Mo Bio Laboratories, Inc., San Diego, CA, United States), and DNA concentrations were measured using a Qubit 2.0 Fluorometer (ThermoFischer Scientific, Waltham, MA, United States). The extracted DNA was sent to the MIT BioMicro Center Core Facility for sequencing. Libraries were prepared using Nextera DNA Library Prep and DNA was sequenced on an Illumina MiSeq or HiSeq 2000 platform at the MIT Center for Environmental Health Sciences (CEHS) Genomics Facilities Core. The sequencing yielded an average of 250 base pair (bp) paired end reads. Quality control was performed using Trimmomatic 0.36 with default parameters and a minimum sequence length of 50 base pairs (Bolger et al., 2014). Reads were assembled using SPAdes 3.9.0 (Bankevich et al., 2012) with a minimum contig length of 1,000 base pairs. Target Cyanobacteria genomes were extracted and binned using sequence composition and read-pair linkage through the CONCOCT algorithm within the Anvi’o software (Alneberg et al., 2014; Eren et al., 2015). MAGs were manually refined and curated using the interactive interface in the Anvi’o program (Eren et al., 2015). After refinement, genome completeness and contamination were assessed using the CheckM workflow (Parks et al., 2015) and output is listed in Supplementary Table 2. Following successful sequencing and reconstruction of cyanobacterial genomes, small and large subunit ribosomal proteins were identified and extracted from the resulting genomic assemblies using BLAST+ with a blastx search of whole genomes against an index of ribosomal proteins.

### Data Deposition

Sequence data for newly sequenced cyanobacteria (Table 1) are available on the NCBI database under accession numbers PVWP00000000, PXOH00000000, PVWE00000000, PVWD00000000, PYGV00000000, PYFY00000000, PYEQ 00000000, PYER00000000, PVWO00000000, PVWN00000000, PVWM00000000, PYCI00000000, PVWL00000000, PVWK 00000000, PVWJ00000000, PVWH00000000, PVWI00000000, PVWG00000000, PVWF00000000, SAMN08828726, and SAMN08828728. All alignment and tree files are available on Figshare at doi: 10.6084/m9.figshare.7629383.

### Phylogenetic Tree Reconstruction

Sequences of large and small subunit ribosomal proteins for cyanobacteria, plastids and bacterial outgroups were aligned with MUSCLE v3.8.31 (Edgar, 2004) and concatenated using FASconCAT v1.0 (Kück and Meusemann, 2010). Substitution model analyses were carried out using ProtTest v. 3.4.2 (Abascal et al., 2005; Darriba et al., 2011).

The monophyly of Cyanobacteria+plastids was tested for each ribosomal gene family by producing maximum-likelihood trees using IQtree (LG model with four gamma distributed rate categories). The resulting trees show that the concatenated alignment does not contain extensive HGT from outside this group that could potentially confound phylogenetic inference. A small number of sequences were identified as placing outside of Cyanobacteria+plastids in their respective gene trees: L15, L18 (*Chlamydomonas reinhardtii*, *Volvox carteri* f. nagariensis); S11 (*Ectocarpus siliculosus*, *Arthrospira*, *Nannochloropsis salina*). It is unclear if these exceptions were due to legitimate HGT detection, or lack of phylogenetic signal within such short individual proteins. The impact of these potentially transferred sequences was assessed by removing them from the concatenated protein alignment and generating a maximum-likelihood tree (IQTree, LG model, 4 gamma distributed rate categories). The resulting placement of plastids within the deep cyanobacterial tree topology was identical to that observed in maximum-likelihood and Bayesian inference consensus trees generated from the full concatenated protein dataset, and so these were retained in subsequent analyses.

Phylogenetic trees were made using both maximum likelihood (ML) criteria with RAxML v8.1.9 (Stamatakis, 2006) [four gamma distributed site rate categories with estimated shape parameter (α = 0.827794) and an LG substitution model], and Bayesian inference using PhyloBayes 3.3 (Lartillot et al., 2013) (C20 site specific substitution models, convergence criterion cutoff of 0.3 for all parameters). Bayesian inference phylogenies were also generated from Dayhoff-recoded alignments to test the potential impact of nucleotide compositional bias on non-synonymous substitutions. Three recordings were performed: (A) the dayhoff6 recoding as included in the PhyloBayes 3.3 package (recoded amino acid sets: AGPST, DENQ, HKR, FYW,ILVM); (B) a more conservative recoding that synonymized only physiochemically similar amino acids with varying G+C content between synonymous codons (recoded amino acid sets: ILVM, FYW, KR, NQ); (C) a highly conservative recoding based on the specific prediction of a high rate of substitution between K and R within plastids driven by nucleotide bias (recoded amino acid sets KR) (Li et al., 2014).

Ribosomal RNA sequence alignments were performed using the Silva SINA (v1.2.11) global aligner service (Quast et al., 2013). The subsequent alignments were manually curated, as several gap-adjacent regions showed clearly misaligned bases. The resulting edited alignments were then further curated by the removal of “gappy” regions that contained ambiguously aligned residues. The unedited, edited, and trimmed alignments, including a list of the removed regions, are available as supporting materials (section “rRNA Sequence Trimming” in Supplementary Methods and Supplementary Table 1). Maximum-likelihood trees for 16S, 23S, and concatenated 16S/23S alignments were generated in IQ-TREE (Nguyen et al., 2015), with best-fitting models determined by ModelFinder (Kalyaanamoorthy et al., 2017). 100 bootstrap replicates were performed in each case. Best-fitting models were determined by Bayesian Information Criterion (BIC). For the 16S and concatenated rRNA alignments, the best fitting model was GTR, with empirical base frequencies and five free rate parameters. For 23S, the best fitting model was SYM with five free rate parameters. Bayesian consensus trees were generated in Phylobayes3.3f (Lartillot et al., 2013), under a CAT-GTR model including site-specific base exchangeabilities, with consensus trees generated from tree samples following convergence in two chains across all parameters to a variance of <0.3.

## Results and Discussion

### Cyanobacterial Tree Topology

The analyses presented here combine the sequences of 37 cyanobacterial and 32 plastid genomes with 20 newly sequenced species to expand cyanobacterial phylogeny and constrain the divergence of plastids with better resolution (Table 1). Of the 20 genomes that our group sequenced for this study, 17 were high quality, and 4 were of medium quality, according to the current accepted standards (Bowers et al., 2017). We conducted both ML and Bayesian analyses that used a concatenation of 30 large and small subunit ribosomal protein sequences (Table 2). We chose to use amino acid sequences rather than nucleotide sequences because the latter are more strongly affected by saturation over long time scales (Li et al., 2014). While Cyanobacteria and plastids share additional homologous proteins, such as those associated with the photosynthetic machinery, ribosomal sequence data remain a standard for species-tree phylogenetic inference for three main reasons. Firstly, ribosomal proteins are generally conserved, and provide many thousands of well-aligned, informative sites. Secondly, ribosomal proteins are infrequently transferred and are usually present as orthologs encoded by single gene loci within genomes. This makes reticulate scenarios that can confound species tree inferences (gene transfers, duplications, and losses) unlikely. Thirdly, the use of ribosomal protein sequences shared by many bacterial phyla enables the rooting of Cyanobacteria by outgroup sequences. This is critical for inferring deep cyanobacterial phylogenetic topology and the inferred placement of plastids (Lyons-Weiler et al., 1998), and cannot be done with sequences that are absent from the bacterial species tree outgroups.

**FIGURE 1:**
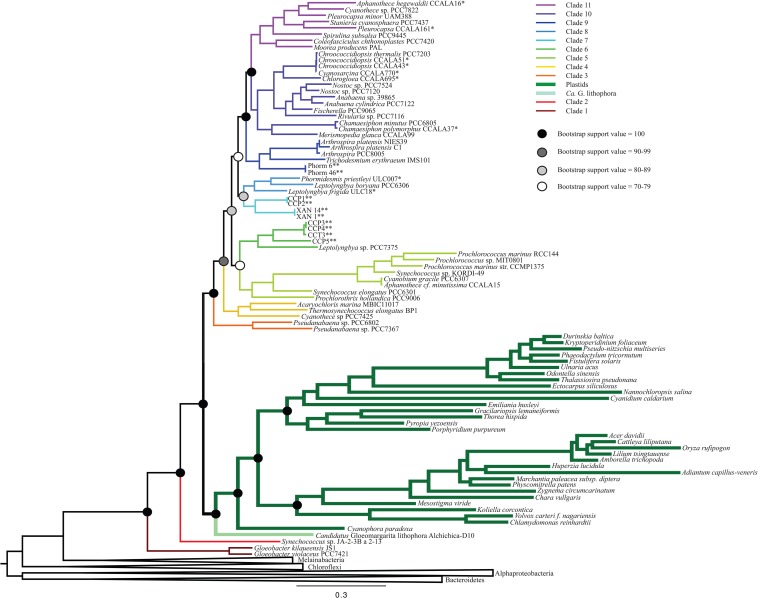
A maximum likelihood (ML) phylogenetic tree made using a concatenation of 30 large and small subunit ribosomal proteins (Table 2) and RAxML. This tree supports a deep placement of the plastid clade with high bootstrap support. Bootstrap values are denoted for major divergences of cyanobacterial and plastid clades. All internal nodes have bootstrap values of >90.

**FIGURE 2:**
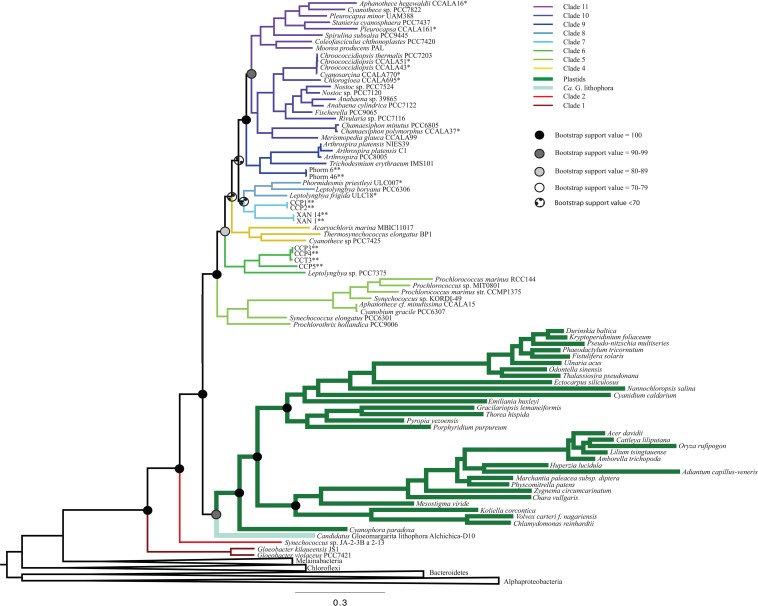
A ML phylogenetic tree made without the inclusion of *Pseudanabaena* using RAxML. The placement of plastids in this tree is consistent with Figure 1, but with lower bootstrap support values. Additionally, the placement of clade 4 is shifted to be more recently branching in this tree than in trees that contain *Pseudanabaena*.

All iterations of our models and tree reconstruction methods recovered congruent sets of bipartitions between deeply branching cyanobacterial taxa including *G. lithophora*, and the plastid donor/ancestor lineage. For non-recoded analyses that included bacterial outgroups for rooting, *Gloeobacter* was the deepest branching group (assigned here as clade 1, including *G. kilaueensis* and *G. violaceus*), followed by a deeply branching strain of *Synechococcus* (*Synechococcus* sp. JA-2-3B a 2-13; clade 2). In all analyses, plastids and *G. lithophora* diverged immediately after *Synechococcus* sp. JA-2-3B a 2-13. These placements were statistically supported with 100% bootstrap support (Figure 1) and posterior probability (Supplementary Figure 1). A clade comprised of two strains of *Pseudanabaena* (sp. PCC7367 and sp. PCC6820, clade 3) did not diverge until after plastids, followed by clade 4, comprised of *Acaryochloris marina* MBIC11017, *Thermosynechococcus elongatus* BP1, and *Cyanothece* sp. PCC7425 (Figure 1 and Supplementary Figure 1). An additional analysis that excluded *Pseudanabaena* recovered a similarly deeply branching plastid clade, although the removal of *Pseudanabaena* altered the cyanobacterial topology slightly with lower bootstrap support (Figure 2) and posterior probability (Supplementary Figure 2) relative to trees that included *Pseudanabaena* (e.g., nodes that had bootstrap support values of 90–99 decreased to well below 70, and those that had posterior probabilities of 1 decreased to 0.8–0.9). Specifically, the removal of this group altered the position of clade 4, placing it between clades 6 and 7 with very low bootstrap support (<30). Because of the low support for this topology, and its inconsistency with previously published trees, we favor the topology that includes *Pseudanabaena*. Notably, this test confirms that the deep placement of plastids in our tree is not driven by the inclusion or exclusion of *Pseudanabaena*.

The remaining cyanobacterial species grouped into seven clades (clades 5 through 11). Two of these clades were new, and five corresponded to clades recovered and described in previously published cyanobacterial phylogenies. Clade 5 includes *Prochlorothrix hollandica* as well as several species of marine *Synechococcus* and *Prochlorococcus*, and corresponds to clade 3 in Schirrmeister et al. (2015) and clade C in Shih et al. (2013). Clades 9, 10, and 11, respectively, correspond to clades 4, 5, and 6 in Schirrmeister et al. (2015) and A, B1, and B2 in Shih et al. (2013). Clades 6 and 7 in our study contain newly sequenced species of benthic cyanobacteria from the Arctic, Cape Cod and Yellowstone National Park. Some of these strains group with *Leptolyngbya boryana* (clade 6) that corresponds to clade D in Shih et al. (2013), while the strains from the Arctic group together into a new clade (clade 8). Our analyses also identified one additional clade that was composed entirely of newly sequenced cyanobacteria, and therefore is not directly comparable to any previously described clades. This clade (clade 7) included four newly sequenced filamentous cyanobacteria enriched from microbial mats (Momper et al., 2019). Other newly sequenced species grouped within the remaining clades in a predictable fashion (i.e., species from culture collections generally grouped with other organisms of the same assigned genus; Figure 1 and Supplementary Figure 1).

**FIGURE 3:**
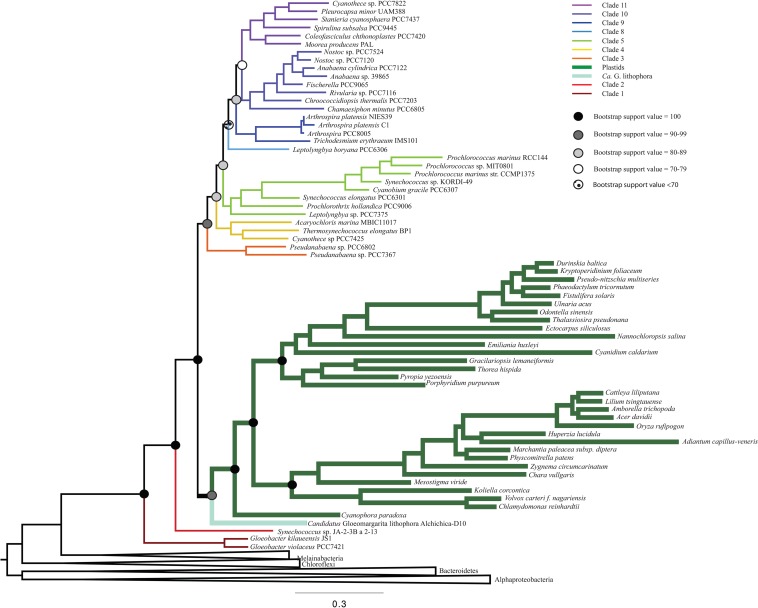
A ML phylogenetic tree that does not include any newly sequenced genomes, made using RAxML. This tree recovers a topology consistent with Figure 1, but with lower bootstrap support values.

To determine the influence of newly sequenced cyanobacterial genomes on the overall topology, we carried out additional analyses that excluded these genomes (Figure 3 and Supplementary Figure 3). These analyses produced a topology consistent with those that include new sequences (Figure 1). However, the trees that did not include the new ribosomal protein sequences had lower bootstrap support values (Figure 3) and posterior probabilities (Supplementary Figure 3) compared to analyses that included newly sequenced cyanobacteria (e.g., some nodes that previously had bootstrap support values of 100 decreased to 70–79, and some nodes that previously had posterior probabilities of 1 decreased to 0.7–0.79). These results support our tree topology and demonstrate the value of including newly sequenced strains in our phylogenetic analyses.

To extend comparisons to previous analyses and further investigate the ribosomal evolutionary signal within plastids, trees were also generated for ribosomal RNA sequences, including 16S, 23S, concatenated 16S and 23S alignment datasets, and ribosomal protein trees for Cyanobacteria alone, without outgroups (Supplementary Figures 6–12). For each dataset, maximum-likelihood and Bayesian inference consensus trees produced similar topologies and placements for plastids and *G. lithophora.* For the 16S dataset, both trees recovered a deep placement of plastids within Cyanobacteria, deeper than *Pseudanabaena*, albeit with low bootstrap and posterior probability support (38/100 and 0.53, respectively). In contrast to trees generated from concatenated proteins, *G. lithophora* grouped more deeply, together with *Synechococcus* sp. JA-2-3B’a(2-13). Support values across internal nodes for shallower cyanobacterial clades were extremely low, and most cyanobacterial groups shared a major multifurcation in the Bayesian inference consensus tree. 23S alignments produced substantially different tree topologies. While plastids grouped together with *G. lithophora* with high bootstrap and posterior probability values (83/100 and 1.00, respectively) similar to the protein trees, this group has a shallow placement within the tree, similar to the position observed in Ochoa de Alda et al. (2014), and with very low bootstrap and posterior probability values (25/100 and 0.67, respectively). Interestingly, in the 23S trees *Pseudanabaena* also branches shallowly in contrast to 16S and ribosomal protein tree results, although still relatively close to *G. lithophora* and plastids. Concatenated 16S and 23S alignments generate trees similar to the 23S tree, with somewhat higher but still relatively low support values for placements of major groups, and a major multifurcation persisting in the Bayesian inference consensus tree.

Ribosomal RNA appears to contain a relatively weak evolutionary signal for resolving the placement of many major groups of cyanobacteria, including plastids and *G. lithophora*. This is similar to the result obtained in previous analyses (e.g., Ponce-Toledo et al., 2017), which also recovered low supports and multifurcations for these clades. However, we also note that the 16S rRNA tree’s “deep” placement of plastids, while poorly supported, does recover as a consensus signal two of the key bipartitions observed in trees generated from ribosomal protein datasets. Clades 1–2, *Gloeomargarita*, and plastids group together to the exclusion of other cyanobacteria, and cyanobacterial clades 4–11 subsequently group together to the exclusion of other cyanobacteria and plastids, including *Pseudanabaena* (Clade 3). The generally low support values and surprisingly inconsistent tree topologies between 16S and 23S datasets suggest that a focus on protein sequence analysis is the potentially more fruitful approach for investigating of plastid origins.

### Testing the Position of Plastids

The placement of plastids within our trees is deeper than the placements that have been suggested by most analyses to date. Our analyses consistently show a deep divergence of the plastid/*G. lithophora* clade after the deeply branching *Synechococcus* sp. JA-2-3B a 2-13, but before clade 3 (*Pseudanabaena*; Figure 1 and Supplementary Figure 1). This bifurcation, supported by high bootstrap values and high posterior probabilities, indicates an ancient ancestry of plastids. Previous studies that place plastids within the nitrogen-fixing cyanobacteria (clades 9 and 10 in this study) recover a shallow placement using predominantly nucleotide sequences or protein sequences from nuclear encoded proteins in photosynthetic eukaryotes and their homologs in cyanobacteria (Table 3). These studies use data sets that range from small subunit rRNA sequences to genes such as *rbcl* (Douglas and Turner, 1991; Falcón et al., 2010) and nuclear encoded proteins from photosynthetic eukaryotes with cyanobacterial homologs (Dagan et al., 2012), and many include only a limited dataset of cyanobacteria. A few analyses using nucleotide data have produced topologies with a deeper placement of plastids (Nelissen et al., 1995; Turner et al., 1999), but these also used a limited set of cyanobacterial sequences and had low bootstrap support values. In contrast, studies based on amino acid sequences generally recover a deeper placement of plastids (Rodríguez-Ezpeleta et al., 2005; Reyes-Prieto et al., 2010; Criscuolo and Gribaldo, 2011; Shih et al., 2013). However, analyses that recovered a deep placement either showed a divergence of plastids after the *Pseudanabaena* (Shih et al., 2013) or did not include deeply branching groups like *Pseudanabaena* and *Synechococcus* sp. JA-2-3B a 2-13 at all (Rodríguez-Ezpeleta et al., 2005; Reyes-Prieto et al., 2010; Criscuolo and Gribaldo, 2011). A recent analysis (Sánchez-Baracaldo et al., 2017) used a large set of concatenated proteins including parts of the photosynthetic machinery, resulting in a placement of plastids after both the filamentous *Pseudanabaena* and the clade containing unicellular *Thermosynechococcus*, *Cyanothece*, and *Acaryochloris* (clade 4 in this study). This study used a technique in which the cyanobacterial topology was produced using concatenated proteins as a fixed “backbone” onto which the plastids were placed based on their aligned gene sequences (Sánchez-Baracaldo et al., 2017). The same study also recovered a broadly similar topology using a concatenation technique without the “backbone” (Sánchez-Baracaldo et al., 2017). In contrast, our analyses use a consistent dataset across taxa and include bacterial outgroups to allow for a true root to be placed on the tree. Using this approach, we recovered a deeper placement than previous analyses (Table 3), before *Pseudanabaena*, with high resolution and support. The same deep cyanobacterial and plastid phylogeny was recovered in the absence of outgroups (Supplementary Figure 12). Support values were slightly lower in these cases, but still high. This is expected, as the presence of outgroups polarizes characters within the tree.

**TABLE 3:** Previous phylogenetic studies of plastid placement within the cyanobacterial tree.

**Study**	**Data Set**	**Type of sequence data**	**Tree reconstruction method**	**Key Taxa**	**Placement of plastids**	**Preceding cyanobacterial group**
Douglas and Turner (1991)	16S rRNA	Nucleotide	Least squares	P, Ps	Shallow	Filamentous cyanobacteria, including Pseudanabaena
Nelissen et al. (1995)	16S rRNA	Nucleotide	Neighbor joining, census maximum parsimony and distance matrix	P, Ps	Deep	Pseudanabaena
Turner et al. (1999)	16S rRNA	Nucleotide	Maximum likelihood	P, Ps	Deep	Pseudanabaena
Rodríguez-Ezpeleta et al. (2005)	Plastid sequence orthologs	Amino acid	Bayesian, maximum likelihood, maximum parsimony	P	Deep	Thermosynechococcus
Deusch et al. (2008)	Nuclear encoded proteins and cyanobacteria homologs	Amino acid	Neighbor joining, Maximum likelihood	P	Shallow	Nitrogen fixing cyanobacteria
Falcón et al. (2010)	16S rRNA and *rbcL*	Nucleotide	Bayesian	P	Shallow	Nitrogen fixing cyanobacteria
Reyes-Prieto et al. (2010)	Plastid sequence orthologs	Amino acid	Maximum likelihood	P	Deep	Synechococcus JA 2 3 Ba
Criscuolo and Gribaldo (2011)	Plastid sequence orthologs	Amino acid	Maximum likelihood	P	Deep	Synechococcus JA 2 3 Ba
Dagan et al. (2012)	Nuclear encoded proteins and cyanobacteria homologs	Amino acid	Neighbor joining/supermatrix, Maximum likelihood supermatrix	P	Shallow	Nitrogen fixing cyanobacteria
Shih et al. (2013)	Conserved cyanobacterial and plastid proteins	Amino acid	Maximum likelihood	P, Ps	Deep	Pseudanabaena
Ochoa de Alda et al. (2014)	Combinations of core cyanobacterial genes and plastid orthologs	Nucleotide	Bayesian	P, Ps	Shallow	Nitrogen fixing cyanobacteria
Li et al. (2014)	Concatenated plastid protein-coding sequences and corresponding amino acid sequences	Amino acid and nucleotide	Bayesian and maximum likelihood with recoding	P	Deep	Synechococcus JA 2 3 Ba
Uyeda et al. (2016)	Concatenated cyanobacterial protein sequences plus two ribosomal RNA sequences	Amino acid	Bayesian and maximum parsimony	Ps	N/A	N/A
Ponce-Toledo et al. (2017)	Plastid protein coding sequences and cyanobacterial orthologs	Amino acid	Bayesian and maximum likelihood	P, Ps, Gl	Deep	Pseudanabaena
Sánchez-Baracaldo et al. (2017)	Conserved cyanobacterial protein coding sequences and plastid orthologs	Nucleotide	Bayesian and maximum likelihood	P, Ps, Gl	Deep	Thermosynechococcus
Walter et al. (2017)	Ribosomal RNA sequences and a group of conserved protein sequences	Amino acid	Maximum likelihood	Ps	N/A	N/A
This study	Conserved ribosomal proteins	Amino acid	Bayesian, maximum likelihood, protein recoding	P, Ps, Gl	Deep	Synechococcus JA 2 3 Ba

*The cyanobacterial clade that diverges immediately before the plastids in each study is listed in the final column. All studies in this table that place the plastids immediately following *Synechococcus* sp. JA-2-3B a 2-13 do not include *Pseudanabaena* in their analyses (Douglas and Turner, 1991; Nelissen et al., 1995; Turner et al., 1999; Rodríguez-Ezpeleta et al., 2005; Deusch et al., 2008; Falcón et al., 2010; Reyes-Prieto et al., 2010; Criscuolo and Gribaldo, 2011; Dagan et al., 2012; Shih et al., 2013; Li et al., 2014; Ochoa de Alda et al., 2014; Ponce-Toledo et al., 2017; Sánchez-Baracaldo et al., 2017). Two additional studies are included that show the deep placement of *Pseudanabaena* in the cyanobacterial tree (Uyeda et al., 2016; Walter et al., 2017).*

A recent study that described a newly sequenced cyanobacterium (*G. lithophora*) suggested that this group is a sister group to the plastids and also recovered a deep divergence of both *G. lithophora* and plastids, after the divergence of *Pseudanabaena* (Ponce-Toledo et al., 2017). However, it is possible that the sister relationship between plastids and *G. lithophora* may reflect a long branch attraction artifact (LBA), or otherwise influence the placement of plastids with respect to other cyanobacterial groups. To test the effect of *G. lithophora* on the placement of the plastids in the cyanobacterial tree, we ran two sets of analyses; one that included *G. lithophora* (Analysis 1, Figure 1 and Supplementary Figure 1) and one that did not (Analysis 2, Supplementary Figure 4). The results confirmed that the placement of the plastids in our topology was not affected by the exclusion or inclusion of *G. lithophora*. Analysis 1 placed *G. lithophora* as a sister group to the plastids, and the rest of the tree topology was identical to the analysis that did not include *G. lithophora* (Analysis 2). Both analyses also recovered identical deep branching of the plastids immediately after clade 2 (Figure 1 and Supplementary Figures 1, 4). Therefore, our analyses support *G. lithophora* as a sister group to the cyanobacterial plastid ancestor, but the inclusion of this group does not impact the placement of plastids within our phylogenetic analyses. Furthermore, the persistent placement of this group on a short internal branch of the tree shows that the observed placement of plastids is not an artifact arising from LBA.

It has been suggested that the reason for the large discrepancy in the placement of plastids may result from the type of sequences used (Li et al., 2014; Ochoa de Alda et al., 2014). Ochoa de Alda et al. (2014) explored this hypothesis by testing multiple datasets and phylogenetic approaches including (1) large and small subunit rRNA sequences with and without removal of saturated sites; (2) a consensus tree of gene trees including both cyanobacteria and plastids; and (3) phylogenetic analysis of concatenated protein alignments. Their results showed a clear difference between the topologies produced by the gene sequences, which recovered a shallow branching of the plastids, and those produced by amino acid sequences, which show a deeper placement (Ochoa de Alda et al., 2014). The authors attributed this discrepancy to differences in model selection rather than the datasets used, and proposed that the CAT model implemented in nucleotide analyses is a better predictor of the evolutionary relationships than the LG + discrete gamma model used in amino acid analyses (Ochoa de Alda et al., 2014). Because of this, the authors favored the shallow placement produced by analyses using core cyanobacterial and plastid genes with a CAT model (Ochoa de Alda et al., 2014). In contrast, our alignment generates the same tree topology under both an LG substitution model and a CAT site-specific substitution model, showing that our result is not sensitive to this particular increase in model complexity.

Li et al. (2014) tested the difference in plastid placement between phylogenies based on nucleotide sequences and those based on the proteins that these nucleotide sequences encode. These authors suggest that the reason for this discrepancy may be related to compositional biases in the first and third codon positions of the nucleotide sequences, which supports the use of amino acid sequences over nucleotide sequences for these types of analyses (Li et al., 2014). The use of non-homogeneous composition models and a codon-degeneracy recoding technique to reconstruct the cyanobacteria/plastid phylogeny in their study results in a tree that is similar to our results, and places the root of the plastids deep within the tree, after *Synechococcus* sp. JA-2-3B a 2-13 (Li et al., 2014). However, this study did not include *Pseudanabaena*. Our analyses are the first to support a deep placement of the plastids between *Synechococcus* sp. JA-2-3B a 2-13 and *Pseudanabaena*, and provide an independent support for the findings of Li et al. (2014) using highly conserved concatenated proteins from an expanded set of cyanobacterial taxa.

Given that nucleotide composition may also impact non-synonymous amino acid substitutions (Li et al., 2014), we performed additional Bayesian phylogenetic reconstructions using Dayhoff-recoded alignments designed to mitigate the impact of this effect. All three implemented Dayhoff recoding schemes returned phylogenies with deep cyanobacterial topologies identical to non-recoded analyses, including the monophyly of *G.lithophora* and plastids, and their placement with respect to other cyanobacteria. Furthermore, with one exception, all of these bipartitions were recovered with posterior probabilities of 100%. The exception to this was the monophyly of plastids and *G. lithophora*, with a posterior probability of only 51% in coding scheme [B]. Coding scheme [B] still placed plastids deeper in the tree than all included cyanobacteria except Gloeobacter, Synechococcus, and *G.lithophora* with 100% posterior probability. Of non-consensus bipartitions, 40% rooted plastids one node shallower than *G. lithophora.*

### Phylogeny of Plastids

Our analyses consistently recover the monophly of plastids. In this clade, the cyanelle of *Cyanophora paradoxa* is the deepest lineage, followed by two additional major clades: clade A includes the green algae and land plants and clade B is comprised of the red algae, dinoflagellates, coccolithophores, and diatoms. Even when the deeply diverging *Cyanophora paradoxa* is removed, our analyses recover the sister relationship between clades A and B (see Supplementary Figure 5). This relationship is in agreement with previously published results that support the monophyly of plastids despite the secondary plastid acquisition that led to groups like dinoflagellates (Cavalier-Smith, 1982; Douglas and Turner, 1991; Turner et al., 1999; Moreira et al., 2000; Nozaki et al., 2003; Palmer, 2003; Rodríguez-Ezpeleta et al., 2005). Some uncertainty remains as to the exact relationships between these nucleocytoplasmic lineages and their secondary (and tertiary) endosymbionts (Keeling, 2009), but these much more recent divergences are not the primary focus of this study.

The phylogenetic trees produced by our analyses also show that the plastid lineages have very long branches compared to other cyanobacteria, with the exception of the long branches within the clade containing *Synechococcus/Prochlorococcus* and their close relatives. The long branches of *Prochlorococcus* have been attributed to their reduced genome sizes and accelerated rates of evolution (Sun and Blanchard, 2014), a potentially analogous situation to that within plastids. The transfer of genes from cyanobacteria into ancestral plastid bearing lineages has been noted and investigated by Martin et al. (2002). These authors identified homologous proteins in *Arabidopsis* and three cyanobacterial genomes to determine the amount of gene transfer that likely occurred, and found that roughly 18% of the total Arabidopsis genome was transferred from a chloroplast (Martin et al., 2002). This study underlines the amount of gene transfer that likely occurred through this endosymbiotic event (Martin et al., 2002), an occurrence which may have similarly diminished the genome of the endosymbiont as these genes were transferred to the host.

## Conclusion

The placement of plastids within a cyanobacterial phylogeny is key to understanding the evolutionary timing and relationship of these groups, but this placement remains in question. Our study generates phylogenies using a concatenation of highly conserved protein sequences from an expanded set of cyanobacterial and plastid lineages. We recover a deeper placement of the plastid/*G. lithophora* clade than has been suggested by most previous studies. This topology is the first to demonstrate a divergence of plastids before the group containing *Pseudanabaena*, after the deeply branching *Synechococcus* sp. JA-2-3B a 2-13. Our results are consistent with the findings of Li et al. (2014) using a highly conserved set of concatenated proteins and expanded set of taxa, and are statistically supported in both maximum-likelihood and Bayesian phylogenetic reconstructions, as well as multiple sequence recoding schemes. Additionally, the trees in this study show very long branches among plastid lineages, consistent with a faster rate of evolution of plastids relative to crown group cyanobacteria. These results are of particular importance for future molecular clock studies of cyanobacterial lineages that include sequences from plastid-containing eukaryotes and fossil calibrations based on these organisms. An accurate tree topology, appropriate modeling of rates of evolution, and well-informed fossil calibrations are all crucial in building molecular clock models to depict the evolutionary history of cyanobacteria and plastids.

## Author Contributions

KM carried out the laboratory culturing and enrichment of environmental samples, data analysis, sequence alignment, phylogenetic tree reconstruction, simplified molecular clock models, and drafted the manuscript. CM participated in sequence collection and analysis. CM, LM, DG, TB, and GF contributed to the manuscript revision and editing. LM carried out the DNA extraction and genome reconstruction of newly sequenced species. DG carried out the statistical analyses. TB and GF conceived and coordinated the study, and provided funding. All authors gave final approval for the manuscript.

## Conflict of Interest Statement

The authors declare that the research was conducted in the absence of any commercial or financial relationships that could be construed as a potential conflict of interest.

## Footnotes

1 https://www.ncbi.nlm.nih.gov/biosample?LinkName=bioproject_biosample_all&from_uid=436169
