# Supplementary material for: An Expanded Ribosomal Phylogeny of Cyanobacteria Supports a Deep Placement of Plastids
Source: Front Microbiol. 2019 Jul 12;10:1612. doi: 10.3389/fmicb.2019.01612 (PMC6640209; doi:10.3389/fmicb.2019.01612)
Supplement: Supplementary file 1 [file Data_Sheet_1.docx]

Supplementary Material

# Supplementary Methods

## Selection of available sequences

We selected a core set of cyanobacterial species, plastid lineages and bacterial outgroups from genomes that were available on the NCBI database (<http://www.ncbi.nlm.nih.gov/>; Table 1). The selection of 36 cyanobacterial species was guided by previous phylogenetic analyses to ensure that all major clades were represented. Four sequences of the newly described Melainabacteria were added as a sister group (Table 1).Thirty two representative plastid lineages were chosen from each of the photosynthetic eukaryotic groups, including glaucophytes, red algae, green algae, land plants, diatoms, dinoflagellates, and haptophytes (Table 1). We chose 45 non-cyanobacterial outgroup bacterial species, including species of Chloroflexi (the anoxygenic phototrophs that contain photosystem II) and a representative set of Bacteroidetes, Alphaproteobacteria, and Chlorobi species (Table 1). The inclusion of outgroup species provided a means of accurately rooting our phylogenetic trees.

## Selection and sequencing of additional strains

We added to our data set 21 previously unsequenced cyanobacteria with unique morphologies, lifestyles or restricted environmental distributions (Table 1). Pure cultures of cyanobacteria with previously unsequenced genomes were ordered from the Culture Collection of Autotrophic Organisms at the Institute of Botany, Czech Republic (CCALA) and the Belgian Co-Ordinated Collections of the University of Liège, Belgium (BCCM/ULC). Environmental samples from the Great Sippewissett Salt Marsh (lower Eastern Buzzards Bay, Cape Cod, MA, USA) and from McMurdo Dry Valley Lakes, Antarctica (generously provided by Dr. Anne Jungblut), were grown and enriched in the laboratory. Sequencing and analyses also included cyanobacteria that were previously enriched from Yellowstone National Park (Bosak et al., 2012).

## rRNA sequence trimming:

23S Gappy removed:

1-6

135-153

286-321

340-536

954-967

1006-1023

1315-1345

1581-1656

1928-1931

1949-1954

1976-2009

2064-2134

2194-2200

2326-2342

2466-2479

2828-2834

3419-3539

3570-3599

3673-3677

16S Gappy removed:

1-10

75-120

207-214

224-286

529-532

1198-1219

1533-1546

# Supplementary Figures and Tables

## Supplementary Figures

**Supplementary Figure 1.** A phylogenetic tree made using Bayesian inference (PhyloBayes) with a C20 model and a concatenation of 30 large and small subunit ribosomal proteins (Table 2). This tree supports a deep rooting of the plastid clade with high posterior probabilities. Posterior probabilities are denoted for major divergences of cyanobacterial and plastid clades. All internal nodes have posterior probabilities of >90.

**Supplementary Figure 2.** A phylogenetic tree made using Bayesian inference (PhyloBayes) with a C20 model without the inclusion of *Pseudanabaena*. The rooting of plastids in this tree is consistent with Figure 1, but with the overall topology has lower posterior probabilities.

**Supplementary Figure 3.** A phylogenetic tree made using Bayesian inference (PhyloBayes) with a C20 model that does not include any newly sequenced genomes. This tree recovers a topology consistent with Figure 1, but with lower posterior probabilities.

A

**B**

**Supplementary Figure 4.** A phylogenetic trees made using ML (A; RAxML) and Bayesian (B; PhyloBayes) models that do not include *G. Lithophora*.

**A**

**B**

**Supplementary Figure 5.** A phylogenetic trees made using ML (A; RAxML) and Bayesian (B; PhyloBayes) models that do not include *Cyanophora paradoxa.*

**Supplementary Figure 6.** 16S rRNA maximum-likelihood tree generated with IQ tree, best fit model (BIC): GTR+F+R5

**Supplementary Figure 7.** 16S rRNA Bayesian consensus tree (Phylobayes, model GTR+CAT)

**Supplementary Figure 8.** 23S rRNA, maximum-likelihood tree (IQ tree, best fit model (BIC): SYM+R6)

**Supplementary Figure 9.** 23S rRNA, Bayesian consensus tree (Phylobayes, model: GTR+CAT)

**Supplementary Figure 10.** 16S+23S concatenated rRNA, maximum-likelihood tree (IQ tree, best fit model (BIC): GTR+F+R5)

**Supplementary Figure 11.** 16S+23S concatenated rRNA, Bayesian concensus tree (Phylobayes, model: GTR+CAT)

A

B

**Supplementary Figure 12.** Phylogenetic trees made using ML (A; RAxML) and Bayesian (B; PhyloBayes) models that excludes outgroups but still shows a deep placement of plastids within the cyanobacterial tree.

## Supplementary Tables:

| **Phylobayes naming** | **taxon** | **Accession** | **16S** | **23S** | **in Concatentation** |
| --- | --- | --- | --- | --- | --- |
| P12 | Cyanidium caldarium | AF022186 | x | x | x |
| C3 | Prochlorothrix hollandica PCC 9006 | ANKN01000130 | x | x | x |
| C25 | Pseudanabaena sp. PCC 6802 | ALVK01000018 | x | x | x |
| P29 | Physcomitrella patens | AP005672 | x | x | x |
| P23 | Emiliania huxleyi | AY741371 | x | x | x |
| P31 | Zygnema circumcarinatum | AY958086 | x | x | x |
| C10 | Cyanothece sp. PCC 7425 | CP001344 | x | x | x |
| C12 | Nostoc sp. PCC 7524 | CP003552 | x | x | x |
| C21 | Pseudanabaena sp. PCC 7367 | CP003592 | x | x | x |
| C27 | Chroococcidiopsis thermalis PCC 7203 | CP003597 | x | x | x |
| C14 | Anabaena cylindrica PCC 7122 | CP003659 | x | x | x |
| C29 | Prochlorococcus sp. MIT 0801 | CP007754 | x | x | x |
| P24 | Thalassiosira pseudonana | EF067921 | x | x | x |
| P20 | Pseudo-nitzschia multiseries | KR709240 | x | x | x |
| P11 | Marchantia paleacea subsp. diptera | LC035012 | x | x | x |
| C30 | Moorea producens PAL | MKZR01000001 | x | x | x |
| P28 | Odontella sinensis | Z67753 | x | x | x |
| C1 | Coleofasciculus chthonoplastes PCC 7420 | ABRS01000062 | x | x | x |
| C5 | Fisherella sp. NIES 3754 | AP017305 | x | x | x |
| P21 | Amborella trichopoda | AJ506156 | x | x | x |
| C17 | Thermosynechococcus elongatus BP-1 | BA000039 | x | x | x |
| C11 | Cyanobium gracile PCC 6307 | CP003495 | x | x | x |
| C2 | Rivularia sp. PCC 7116 | CP003549 | x | x | x |
| C16 | Stanieria cyanosphaera PCC 7437 | NC_019748.1 | x | x | x |
| C22 | Gloeomargarita lithophora Alchichica-D10 | CP017675 | x | x | x |
| P3 | Chara vulgaris | DQ229107 | x | x | x |
| P25 | Kryptoperidinium foliaceum | GU591328 | x | x | x |
| P16 | Nannochloropsis salina | KC598088 | x | x | x |
| P8 | Cyanophora paradoxa | KM198929 | x | x | x |
| P9 | Cattleya liliputana | KP202881 | x | x | x |
| C32 | Arthrospira platensis C1 | AFXD01000008 | x | x | x |
| C33 | Spirulina subsalsa PCC 9445 | ALVR01000007 | x |  |  |
| P2 | Fistulifera solaris | AP011960 | x | x | x |
| P13 | Adiantum capillus-veneris | AY178864 | x | x | x |
| C31 | Gloeobacter violaceus PCC 7421 | BA000045 | x | x | x |
| C7 | Arthrospira sp. PCC 8005 |  | x | x | x |
| C20 | Gloeobacter kilaueensis JS1 | CP003587 | x | x | x |
| C28 | Synechococcus sp. KORDI-49 | CP006270 | x | x | x |
| P15 | Ectocarpus siliculosus | FP102296 | x | x | x |
| P6 | Durinskia baltica | GU591327 | x | x | x |
| P10 | Thorea hispida | KX284714 | x | x | x |
| P1 | Mesostigma viride | AF166114 | x | x | x |
| C24 | Leptolyngbya sp. PCC 7375 | ALVN01000001 | x | x | x |
| P22 | Pyropia yezoensis | AP006715 | x | x | x |
| C4 | Synechococcus elongatus PCC 6301 | AP008231 | x | x | x |
| C15 | Arthrospira platensis NIES-39 | AP011615 | x | x | x |
| P30 | Porphyridium purpureum | AP012987 | x | x | x |
| C6 | Nostoc sp. PCC 7120 | BA000019 | x | x | x |
| C8 | Synechococcus sp. JA-2-3B'a(2-13) | CP000240 | x | x | x |
| C26 | Trichodesmium erythraeum IMS101 | CP000393 | x | x | x |
| C9 | Acaryochloris marina MBIC11017 | CP000828 | x | x | x |
| C19 | Cyanothece sp. PCC 7822 | CP002198 | x | x | x |
| P4 | Phaeodactylum tricornutum | EF067920 | x | x | x |
| P26 | Ulnaria acus | JQ088178 | x | x | x |
| P17 | Lilium tsingtauense | KM103365 | x | x | x |
| P18 | Koliella corcontica | KM462874 | x | x | x |
| P19 | Gracilariopsis lemaneiformis | KP330491 | x | x | x |
| P27 | Acer davidii | KU977442 | x | x | x |
| P7 | Oryza rufipogon | JN005832 | x | x | x |
| P5 | Volvox carteri f. nagariensis | GU084820 | x | x | x |
| P14 | Chlamydomonas reinhardtii | BK000554 | x | x | x |
| C34 | AE_1 |  | x | x | x |
| C35 | AE_14 |  | x | x | x |
| C36 | T2C2_02 |  | x | x | x |
| C37 | TF_M1_03 |  | x | x | x |
| C38 | ULC_007 |  | x | x | x |
| C13 | Pleurocapsa sp. PCC 7327 | CP003590 | x | x | x |
| C18 | Prochlorococcus marinus MIT9313 | BX548175 |  | x | x |
| C23 | Arthrospira sp. TJSD091 | LAYT01000292 | x |  |  |
| C39 | XAN_14 |  | x |  |  |
| C40 | XAN_1 |  | x |  |  |
| C42 | CCALA_695 |  | x |  |  |
| C41 | CCALA_37 |  | x | x | x |

**Supplementary Table 1.** 16S and 23S rRNA analysis sequence list

| Assembly name | Taxon name or preleminary ID | Completeness | Contamination | MISAG/MIMAG quality |
| --- | --- | --- | --- | --- |
| GCF_003003925.1 | Aphanothece cf. minutissima CCALA 015 | 100 | 0.27 | high |
| GCF_003003775.1 | Merismopedia glauca CCAP 1448/3 | 99.66 | 0.89 | high |
| GCF_003022385.1 | filamentous cyanobacterium CCP3 | 99.65 | 2.01 | high |
| GCF_003007785.1 | Aphanothece hegewaldii CCALA 016 | 99.49 | 0 | high |
| GCF_003003695.1 | Phormidesmis priestleyi ULC007 | 99.29 | 0 | high |
| SAMN08828726 | XAN_1 | 99.29 | 8.07 | medium |
| GCF_003004015.1 | Cyanosarcina cf. burmensis CCALA 770 | 99.26 | 1.78 | high |
| GCF_003003795.1 | Leptolyngbya frigida ULC18 | 99.06 | 0.55 | high |
| SAMN08828728 | XAN_14 | 98.82 | 4.84 | high |
| GCF_003015105.1 | Chroococcidiopsis sp. CCALA 051 | 98.81 | 1 | high |
| GCF_003003885.1 | Chlorogloea sp. CCALA 695 | 98.78 | 0.33 | high |
| GCF_003003995.1 | Pleurocapsa sp. CCALA 161 | 98.25 | 1.16 | high |
| GCF_003017855.1 | filamentous cyanobacterium CCT1 | 97.6 | 4.66 | high |
| GCF_003003725.1 | filamentous cyanobacterium Phorm 6 | 97.3 | 0.54 | high |
| GCF_003003615.1 | filamentous cyanobacterium CCP2 | 96.19 | 3.85 | high |
| GCF_003018975.1 | filamentous cyanobacterium CCP4 | 94.07 | 4.08 | high |
| GCF_003003845.1 | Chamaesiphon polymorphus CCALA 037 | 92.52 | 2.04 | high |
| GCF_003003835.1 | Chroococcidiopsis cubana CCALA 043 | 91.7 | 1.7 | high |
| GCF_003017785.1 | filamentous cyanobacterium CCP5 | 89.75 | 7.41 | medium |
| GCF_003003715.1 | filamentous cyanobacterium Phorm 46 | 85.04 | 1.17 | medium |
| GCA_003003915.1 | filamentous cyanobacterium CCP1 | 54.62 | 1.53 | medium |
| Completeness and contamination are reported as % of 100 |  |  |  |  |
| Quality assessment based on standards published in Bowers et al., 2017 |  |  |  |  |

**Supplementary Table 2.** Metagenomic assembly completeness, contamination and quality scores
